# Community Biomass and Bottom up Multivariate Nutrient Complementarity Mediate the Effects of Bioturbator Diversity on Pelagic Production

**DOI:** 10.1371/journal.pone.0044925

**Published:** 2012-09-12

**Authors:** Adriano Caliman, Luciana S. Carneiro, João J. F. Leal, Vinicius F. Farjalla, Reinaldo L. Bozelli, Francisco A. Esteves

**Affiliations:** 1 Departamento de Ecologia, Instituto de Biologia, Universidade Federal do Rio de Janeiro, CCS, Cidade Universitária, Rio de Janeiro, Rio de Janeiro, Brazil; 2 Centro Federal de Educação Tecnológica de Química, Nilópolis, Rio de Janeiro, Rio de Janeiro, Brazil; 3 Núcleo de Pesquisas em Ecologia e Desenvolvimento Sócio Ambiental de Macaé, Rodovia Amaral Peixoto, Macaé, Rio de Janeiro, Brazil; University of Southampton, United Kingdom

## Abstract

Tests of the biodiversity and ecosystem functioning (BEF) relationship have focused little attention on the importance of interactions between species diversity and other attributes of ecological communities such as community biomass. Moreover, BEF research has been mainly derived from studies measuring a single ecosystem process that often represents resource consumption within a given habitat. Focus on single processes has prevented us from exploring the characteristics of ecosystem processes that can be critical in helping us to identify how novel pathways throughout BEF mechanisms may operate. Here, we investigated whether and how the effects of biodiversity mediated by non-trophic interactions among benthic bioturbator species vary according to community biomass and ecosystem processes. We hypothesized that (1) bioturbator biomass and species richness interact to affect the rates of benthic nutrient regeneration [dissolved inorganic nitrogen (DIN) and total dissolved phosphorus (TDP)] and consequently bacterioplankton production (BP) and that (2) the complementarity effects of diversity will be stronger on BP than on nutrient regeneration because the former represents a more integrative process that can be mediated by multivariate nutrient complementarity. We show that the effects of bioturbator diversity on nutrient regeneration increased BP via multivariate nutrient complementarity. Consistent with our prediction, the complementarity effects were significantly stronger on BP than on DIN and TDP. The effects of the biomass-species richness interaction on complementarity varied among the individual processes, but the aggregated measures of complementarity over all ecosystem processes were significantly higher at the highest community biomass level. Our results suggest that the complementarity effects of biodiversity can be stronger on more integrative ecosystem processes, which integrate subsidiary “simpler” processes, via multivariate complementarity. In addition, reductions in community biomass may decrease the strength of interspecific interactions so that the enhanced effects of biodiversity on ecosystem processes can disappear well before species become extinct.

## Introduction

A growing number of evidences in the literature have shown that biodiversity loss can affect the functioning of natural ecosystems [1]–[6]. However, the number of biodiversity and ecosystem function (hereafter BEF) studies is highly uneven relative to the biological community, ecosystem processes and trophic level investigated [7]. Most BEF studies have focused on a single ecosystem process, which frequently represents resource capture or production of biomass within trophic levels (*e.g*., terrestrial primary productivity) or the flow of matter between adjacent trophic levels (*e.g.,* herbivory) in a particular habitat [8]–[11]. Less attention has been devoted to evaluate how multiple ecosystem processes driven by non-trophic interactions among mobile fauna (*e.g.,* ecosystem engineering) indirectly control the rates of processes performed by ecological communities inhabiting different habitats and ecosystems [12]–[14]. In addition, although density-dependent interactions affect per capita and population species resource consumption [15] with consequences for resource partitioning by species and the occurrence of complementary effects of biodiversity [16]–[19], we know very little about whether and how density-dependent effects and species diversity interactively affect ecosystem multifunctionality via non-consumptive biogeochemical interactions (but see [20]).

Because studies have focused on single ecosystem processes, we have neglected the role of diversity in integrated and multiple ecosystem functions [6], [9], [21], but see [22]–[25]. The emphasis on single processes prevents us from proposing and testing new mechanisms that may explain why biodiversity effects often vary across ecosystem processes [26], [27]. Even more unfortunately, the emphasis on single processes has limited our ability to identify novel mechanisms through which biodiversity may directly and indirectly control ecosystem functioning, especially if strong interactions exist among key processes [28]. For example, a study that analyzed the effects of seaweed diversity on the uptake of different forms of inorganic nitrogen showed that if individual species have dominant effects (*i.e.*, sampling effects) on the uptake of particular nitrogen forms, species-rich assemblages may enhance the uptake of bulk inorganic nitrogen through multivariate nutrient complementarity [29]. Such a result raises important questions germane to the BEF debate. One such question involves the possibility of classifying ecosystem processes in a hierarchical conceptual framework to predict the strength of biodiversity effects by considering the number of potential direct and indirect effects that species and their interactions have on ecosystem processes. Integrative ecosystem processes that depend on higher-order additive or non-additive interactions among multiple processes should tend to be more strongly affected by multivariate complementarity and should consequently be more sensitive to variation in the number of species. In contrast, ecosystem processes that are dominantly governed by a particular species should be more dependent on species composition [29]–[31]. It is unclear, however, whether multivariate complementarity effects may transcend trophic levels and habitats by connecting spatially segregated communities.

Bioturbation, the biological reworking of soils and sediments, has been recognized as an archetypal example of ecosystem engineering, modifying physical habitat properties and resource availability to other species [32]–[34]. In aquatic ecosystems, bioturbation by benthic invertebrates is a key process altering microbial community structure and geochemical gradients of sediments and regenerating multiple nutrients across the benthic-pelagic interface [34]–[38]. The cross-habitat nutrient regeneration mediated by bioturbation may be highly important for subsidizing autotrophic and heterotrophic pelagic production [39], [40] because it may affect the rates and ratios of the release of limiting nutrients from the sediment to the water [41]. Although empirical and theoretical studies conducted in marine and freshwater systems have demonstrated that the biodiversity of sediment bioturbators enhances the fluxes of dissolved nutrients from the sediment to the water [42]–50, no study to date has tested whether and how these enhancing effects of biodiversity on the regeneration of benthic-pelagic nutrients propagate to affect the flux of energy and matter across pelagic food webs.

We conducted a laboratory experiment to examine whether and how cross-habitat nutrient regeneration mediated by non-trophic bioturbational interactions and their subsequent effects on pelagic microbial production are affected by community biomass, species richness and the composition of benthic invertebrate ecosystem engineers. We hypothesized that (1) because the density of invertebrate bioturbators is well known to affect benthic-pelagic processes [41], [51], it is probable that biodiversity effects on multiple ecosystem processes vary according to invertebrate community biomass; and (2) because biomass production is an integrative process that depends on the availability of multiple limiting resources, which individually may be more dependent on the effects of species identity than species number, the effects of bioturbator species richness will be stronger on BP than on individual nutrient fluxes due to multivariate nutrient complementarity.

## Methods

### Ethics Statement

The experiment was carried out in accordance with Brazilian regulatory standards for animal ethics, and was approved by the Federal University of Rio de Janeiro and Federal University of Rio Grande do Norte Animal Welfare Units. No licenses were necessary for the collection of animals, because species are widely distributed in South America and commonly found even in highly impacted urban aquatic ecosystems such as the sampling site.

### Study Area and Sampling

Sediment and benthic invertebrates were collected near the littoral region of Imboassica lagoon (lat 22°50'S, long 44°42'W), a tropical, shallow, coastal freshwater ecosystem located in Rio de Janeiro State, Brazil [52]. Untreated domestic sewage input adds large loads of N and P to the lagoon and causes eutrophication [53]. The sediment at the sampling site is predominantly silt and clay with mean total C, N and P concentrations of 11.28 mg/g, 2.12 mg/g and 0.067 mg/g, respectively [54].

Samples from the upper layer of the sediment (0–5 cm) were taken with a core sampler, sieved through 1-mm mesh to remove the macrofauna, frozen for 2 weeks, and then thawed. This procedure removed all metazoans as well as their resistant forms [42]. The azoic sediment was homogenized and allowed to settle for 10 d in a 30-L aquarium with a 10-cm deep layer of filtered (25-µm mesh) lagoon water to reduce the natural heterogeneity of the sediment and to permit recovery of its biogeochemical depth gradient [43,e.g., 55]. The aquarium was aerated constantly and was kept in the dark to prevent primary production.

Individuals of 3 species – larvae (0.7–0.9 cm long) of *Chironomus* sp. (Meigen) (Diptera: Chironomidae), adults (2–3 cm long) of *Heteromastus similis* (Southern) (Polychaeta: Captellidae), and adults (0.3–0.4 cm long) of *Heleobia australis* (D’Orbigny) (Gastropoda: Hydrobiidae) – were collected from the field 1 d before the experiment began and were conditioned in aerated species-specific aquaria filled with lagoon sediment and water to allow them to acclimatize to laboratory conditions. The three species coexist locally [56] and regionally [57] in coastal lakes across southeast Brazil and are major contributors to the total benthic invertebrate biomass in Imboassica lagoon [58]. These species clearly differ in their functional bioturbating activities. These differences can be attributed to differences in the species’ foraging behavior [59] and in their spatial distribution within the sediment [44] (Figure S1).

*Chironomus* sp. is a filter feeder and a tube-dweller. It promotes a continuous water flux through permanent U-shaped burrows, oxygenating the deep layers of the sediment and pumping large amounts of dissolved and particulate material from the sediment to the overlying water [60], [61]. *Heleobia australis* is a surface deposit feeder that plows the surface of the sediment [59]. The species has little effect on vertical sediment geochemistry but can greatly affect interfacial biogeochemical kinetics [43], [44]. *Heteromastus similis* is a head-down subsurface deposit feeder that builds extensive semipermanent galleries in the sediment. It modifies the distribution of sediment organic matter and intensifies benthic-pelagic coupling by sediment advection and upwelling egestion of fecal pellets [62].

### Experimental Design

Benthic invertebrate species richness and composition (1–3 species in all possible combinations, encompassing 7 community treatments) were manipulated across 3 invertebrate biomass levels (150, 300, and 450 mg wet mass) in a full factorial-design replacement series [63] in experimental chambers containing a sediment–water interface (see *Experimental setup, sampling procedures and analyses of response variables* below). Thus, the contribution of a species to total invertebrate biomass was decreased to ½ in 2-species mixtures or to ⅓ in 3-species mixtures compared with its own monocultures. Experimental chambers without macrofauna were established as controls and used to estimate the nutrient flux across the sediment–water interface in the absence of benthic invertebrates. All macroinvertebrate treatments and controls were replicated 4 times for a total of 88 experimental units. This experimental design allowed us to evaluate whether and how community biomass and bioturbator diversity interact to modify the magnitude of cross-habitat nutrient recycling rates and pelagic microbial productivity across a natural range of species–biomass distributions in Imboassica lagoon.

### Experimental Setup, Sampling Procedures and Analyses of Response Variables

A few hours before starting the experiment, experimental chambers were established by transferring the stabilized sediment into Plexiglas® tubes (20 cm long × 5 cm internal diameter) to a depth of 5 cm. This depth is sufficient to accommodate the natural vertical distribution of these species within the sediment [44]. The overlying water (10-cm depth) of each microcosm was drained and gently replaced by fresh 0.7-µm filtered (GF/F Whatman) lagoon water to reduce nutrients resulting from dead organic material within the sediment and possible planktonic organisms. Individuals of a given species and size were gently collected by sieving the sediment from the particular species-specific aquarium, rinsed to remove attached sediment, weighed to the nearest 0.1 mg (wet mass after blotting excess water) and immediately distributed into the prepared experimental chambers. Therefore, the community and species biomasses were proportional to community and species densities. Throughout the experiment, each experimental chamber was supplied with its own aerator, placed at a room temperature of 25°C and kept in the dark to prevent depletion of dissolved O_2_, stratification of chemicals within the water column, and photosynthesis.

For nutrient analysis, water samples (20 mL) were taken from each experimental chamber at the beginning and the end (48-h incubation) of the experiment, filtered through 0.7-µm pore filters (GF/F Whatman), and immediately frozen until the determination of ammonium (NH_4_-N), nitrate (NO_3_-N) and total dissolved phosphorus (TDP). We evaluated the effects of bioturbator diversity and biomass on these nutrients because they have been demonstrated to be important for subsidizing secondary microbial production in aquatic ecosystems worldwide [64], [65]. The NH_4_-N determination was performed manually with the phenol-hypochlorite technique according to Solorzano [66]. The NO_3_-N concentration was determined by nitrate reduction in a cadmium column with post-nitrite determination by flow injection analysis according to Golterman et al. [67]. Dissolved inorganic nitrogen (DIN) was then calculated by summation of the NH_4_-N and NO_3_-N concentrations in each experimental chamber. The TDP concentrations were measured by autoclaving water samples with potassium persulfate oxidant and analyzing the digested samples photometrically using the ascorbic acid method according to Golterman et al. [67].

The DIN and TDP fluxes for each experimental chamber were primarily calculated as the difference between the initial (*Ci*) and final (*Cf*) DIN and TDP concentrations (µM m^−2^ h^−1^ ), corrected for the volume (*v* ≈ 0.195 L) of the overlying water, the area (*a* = 0.0019 m^2^) of the sediment surface and the incubation time (*t* = 48 h) according to the following equation:
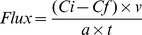



The bioturbation-mediated fluxes of DIN and TDP were then estimated by subtracting the DIN and TDP fluxes quantified in the individual experimental chambers containing fauna from the average DIN and TDP fluxes calculated from the defaunated controls, respectively.

Bacterial production (BP) was estimated based on _3_H-Leucine incorporation into DNA [68]. The BP values were obtained by incubating a 1.3-mL sample of unfiltered water collected from each experimental chamber at the end of the experiment. BP incubations were conducted in the dark for 45 min with 0.1 mL of _3_H-Leucine (5-fold diluted solution, 159 Ci mmol21, Amersham) with a final concentration of 10 nM. After incubation, 90 mL of 100% trichloroacetic acid (TCA) was added to halt the reaction. Each tube was washed sequentially with 5% TCA and 80% ethanol, and 500 mL of Scintillation Cocktail (Aquasol 2, Dupont) was added to each tube. The activity (disintegration per minute, DPM) was measured in a Beckman LS-5600 Liquid Scintillation System. The BP was calculated assuming an intracellular leucine dilution factor of 2 and a cellular carbon-to-protein ratio of 0.86 [69]. The bioturbation-mediated BP was estimated by subtracting the BP quantified in the individual experimental chambers containing fauna from the average BP observed in the defaunated controls.

### Data Analysis

We used two different analyses of variance and least squares linear regressions to investigate the effects of bioturbator biomass, species richness and composition on DIN and TDP fluxes and on BP. We first tested the significance of the effects of bioturbator diversity, biomass (fixed factors) and their interaction on the response variables with a two-factor analysis of variance (two-way ANOVA). We tested the overall effects of species composition (fixed factor) on the 3 measured response variables through a separate nested analysis of variance (nested ANOVA) followed by a Tukey pairwise comparison test, with species composition nested within species richness levels (i.e., 1 and 2). We then regressed DIN and TDP fluxes and BP from each individual experimental chamber as a function of bioturbator species richness (irrespective of biomass) to detect whether the response variables varied monotonically with species richness. We chose to combine these two statistical approaches to analyze our data instead of running a unique complete ANOVA model because the combined analysis better explores the quantitative (species richness) and qualitative (species composition) effects of invertebrate biodiversity on ecosystem processes [70] and provides better insight into the general shape (linear, asymptotic, idiosyncratic) of the diversity-function relationship (see [71] for further details). Prior to statistical analysis, we confirmed the assumptions of homogeneity of residuals (for linear regression) and homogeneity of variances (for factorial and nested ANOVAs) both by regressing the residual values from each response variable on the respective estimated values and by comparing the variance between groups with Bartlett’s test [72]. All analyses were performed using STATISTICA (version 7.0; StatSoft, Tulsa, Oklahoma). The results were considered significant if *p*<0.05.

We quantified diversity “effect sizes” to evaluate how the effects of bioturbator species richness varied along biomass levels and ecosystem processes. We computed the effect sizes with the log response ratio, defined as the natural logarithm (ln) of the treatment mean divided by the control mean [73]. The log response ratio is the most widely used metric for calculating effect sizes and is very intuitive for estimating the proportional difference between treatments. This ratio has sampling properties that are known to be normal and that are robust to bias from small sample sizes [73]. It is also very appropriate for estimating effect sizes in biodiversity and ecosystem function studies [2]. Because it can quantify the proportional difference between the mean value of a species mixture and that of the best constituent monoculture, it is a measure analogous to the *D_max_* index. This index is the most conservative and widely used metric to test the occurrence of transgressive overyielding (i.e., complementarity effects) when the contribution of the component species to the aggregate community process in a species mixture cannot be calculated [74]. Therefore, our diversity effect sizes were quantified as *LR_trans_* (*p*/*m*), where *p* is the observed response in the multispecies treatment (2- and 3-species mixtures) and *m* is the observed response of the best constituent monoculture. We calculated two classes of effect sizes according to our hypothesis. First, we estimated the individual diversity effect sizes (weighted according to the error and sample size for each treatment) for all 2- and 3-species mixtures for each ecosystem process and for each biomass level. To test how the diversity effects on multiple ecosystem processes varied as a function of invertebrate biomass, the overall cumulative effect sizes (after [75]) were then calculated for each biomass level, integrating all diversity treatments and ecosystem processes. This overall cumulative effect size gives a standardized measure of aggregated effects of biodiversity similar to the standardized average of multiple processes commonly used in previous BEF studies [22], [31], [76]. Second, we estimated overall diversity effect sizes relative to each ecosystem process by integrating the individual effect sizes for all biomass and diversity treatments. All cumulative effect sizes and their respective 95% confidence intervals (±95% CI) were calculated with the bootstrapping technique with 9999 iterations. Effect sizes were considered statistically significant if the ±95% CI did not include the value zero. All effect size calculations were performed using MetaWin v. 2.0 [77].

We used generalized linear models (GLM) to test the effects of bioturbation-mediated nutrient fluxes (DIN or TDP) on BP. To select the best approximating model for our data, we calculated the Akaike Information Criterion (AIC*c*, the second-order AIC, necessary for a small sample size). As recommended by Burnham and Anderson [78], AIC*c* differences (Δ*i*) were calculated over all 3 possible candidate models in the set. Because model plausibility decreases with increasing Δ*i*, this quantity represents the level of empirical support for a given model. Finally, Δ*i* values were also used to compute the Akaike weight for each model (ω*i*), which provides evidence that the model is actually the best explanatory model. The software SAM (Spatial Analysis in Macroecology) v4.0 for windows [79] was used to perform the GLM analysis.

## Results

**Figure 1:**
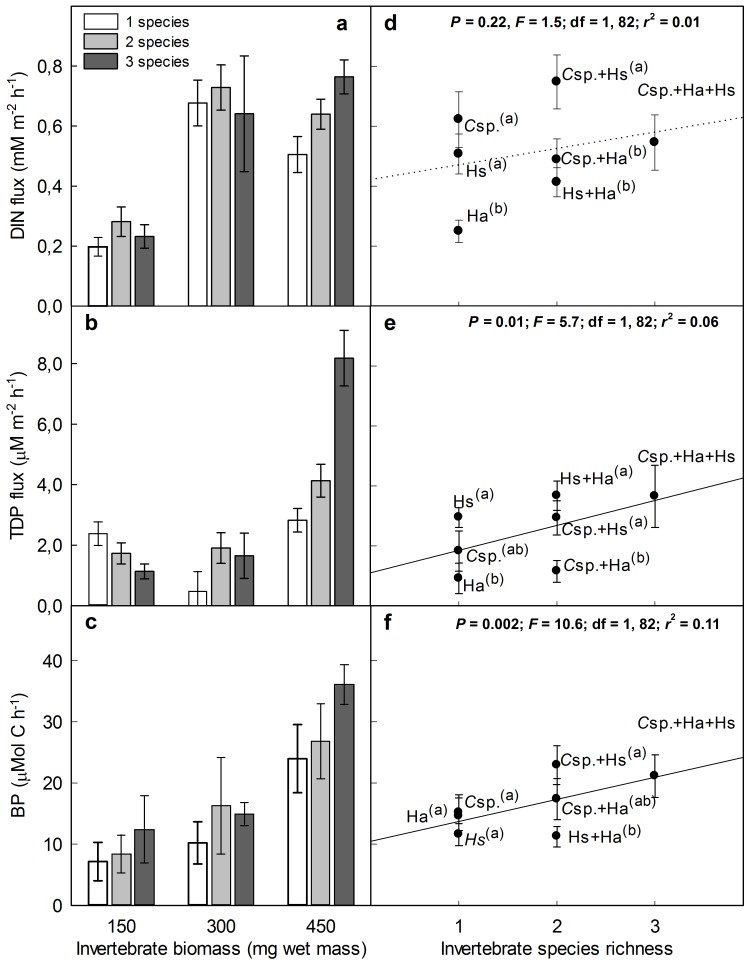
Effects (mean ±1 SE) of invertebrate biomass, species richness and taxonomic composition on (a, d) dissolved inorganic nitrogen (DIN) flux, (b, e) total dissolved phosphorus (TDP) flux and (c, f) bacterioplankton production (BP). Left-hand panels show the interactive effects of invertebrate biomass and species richness on ecosystem processes. Right-hand panels show the overall effects (irrespective of biomass) of species richness and composition (nested factor) on the magnitude of ecosystem processes analyzed. The overall linear effect of species richness was calculated by regressing ecosystem process data from all individual microcosms (*n* = 84, controls not included) as a function of species richness across all biomass levels. The overall effects of taxonomic composition are shown by nested comparisons among mean values across all biomass levels for each individual species and 2-species mixtures. Treatments marked with different letters within the same species richness level differ significantly from one another (Tukey test, *P*<0.05). *C*sp. = *Chironomus* sp., *Hs* = *Heteromastus similis*, *Ha* = *Heleobia australis*.

Neither invertebrate species richness nor its interaction with biomass had statistically significant effects on the DIN flux (Fig. 1a; Table 1). The variation in DIN flux was best explained statistically by the individual effects of biomass and species composition (Fig. 1a,d; Table 1). In general, the DIN flux appeared to be driven primarily by *Chironomus* sp., as shown by the highest values of DIN flux in the treatments containing this species (Fig. 1d). The TDP flux, however, was significantly affected by all of the manipulated experimental factors (Table 1). Invertebrate biomass was the most important factor explaining the TDP flux and also significantly changed the effect of the bioturbator species richness on TDP flux, as indicated by the significant interaction between them (Table 1). This interaction was most evident at the highest biomass level (450 mg), where the TDP flux in the 3-species mixture was approximately 150% and 99% higher than the averages of the monocultures and of the 2-species mixtures, respectively (Fig. 1b). Linear regression analysis showed that species richness also had a positive and significant linear effect on the TDP flux (Fig. 1e), representing, on average, a release of 0.83 µM m^−2^ h^−1^ of TDP from the sediment to water for each species addition. However, as indicated by the significant effect of species composition (Table 1), a considerable proportion of the effect of species richness on TDP flux should be also attributed to the presence of the species *H. similis* (Fig. 1e). In contrast to the results for the DIN and TDP fluxes, invertebrate species richness was the most important factor for explaining BP, as shown by the occurrence of the highest mean squared values in association with this term (Table 1). Although the individual effect of biomass was also significant, biomass did not interact significantly with species richness. The absence of interaction between these terms was determined by the general consistent effect of invertebrate species richness on BP along the three biomass levels (Fig. 1c). As observed for the TDP flux, the significant effect of invertebrate species richness on BP was also linearly positive, but species richness explained almost two times more variation for BP than for TDP (Fig.1f). The effect of species composition on BP was marginally significant (Table 1) and was determined by the differences among the 2-species assemblages. This result indicated that, in contrast to the findings for the DIN and TDP fluxes, the effects of species identity on BP were very weak (Fig. 1f).

**Table 1:** Summary of the factorial (for species richness and biomass) and nested (for species composition) analyses of variance (ANOVA) for nutrient fluxes and bacterioplankton production.

Source of Variation	df	Mean square	*F*-value	*P*-value
*Flux of dissolved inorganic nitrogen*				
Species richness (*S*)	2	81267	1.86	0.16
Biomass (*B*)	2	1294465	29.70	**<0.0001**
* S*×*B*	4	35807	0.82	0.51
Error	75	43584		
Composition[(S)]	4	403652	6.81	**0.0001**
Error	66	59250		
*Flux of total dissolved phosphorus*				
Species richness (*S*)	2	14.56	5.41	**0.006**
Biomass (*B*)	2	88.97	33.05	**<0.0001**
* B*×*S*	4	18.83	6.99	**<0.0001**
Error	75	2.69		
Composition[(S)]	4	16.28	5.38	**0.0008**
Error	66	3.02		
*Bacterioplankton production*				
Species richness (*S*)	2	5.43	474.40	**<0.0001**
Biomass (*B*)	2	0.63	6.52	**0.002**
* S*×*B*	4	0.01	1.32	0.26
Error	75	0.01		
Composition[(S)]	4	0.06	2.52	**0.048**
Error	66	0.02		

The analyses were performed independently considering invertebrate biomass and invertebrate richness as fixed orthogonal factors, whereas invertebrate species composition was nested under invertebrate species richness irrespective of biomass. Brackets indicate the nesting factor. Bold *P*-values indicate a statistically significant effect (*P*<0.05).

**Figure 2:**
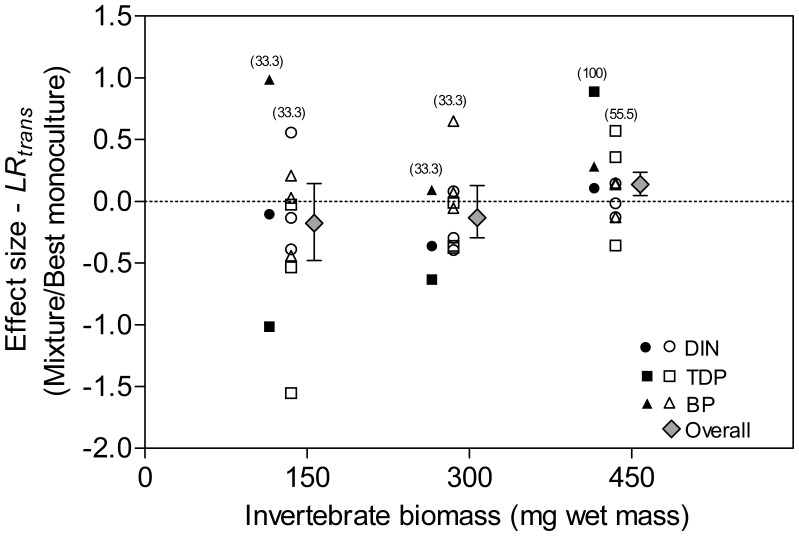
Transgressive overyielding for the ecosystem processes analyzed as a function of invertebrate biomass. Diversity ‘effect sizes’ (standardized ln response ratios) were estimated for individual ecosystem processes for each biomass level by comparing the proportional response of 2- (open symbols) and 3-species mixtures (filled symbols) to their respective best constituent monoculture. Overall cumulative effect sizes (gray diamonds) and their ±95% bootstrapped CI’s were calculated from the weighted integration of the individual effect sizes calculated for all combined ecosystem processes and species richness treatments throughout 9999 iterations. Significant overall transgressive overyielding occurs if the value of *LR_trans_* and its confidence interval are greater than zero (dashed line). Numbers in parentheses represent the proportions of treatments with *LR_trans_* >0 for the respective invertebrate biomass and species richness levels. Abbreviations for ecosystem processes are as in figure 2.

The occurrence of complementarity effects, as indicated by positive *LR_trans_* values, occurred in 44% of all possible (16 out of 36) comparisons calculated between species mixtures and their respective best constituent monoculture. However, both the frequency of positive effect sizes and the magnitude of the overall cumulative effect sizes were higher at the highest invertebrate biomass level. This result indicates that the effects of bioturbator diversity on an aggregate measure including multiple ecosystem processes are stronger as the community biomass increases (Fig. 2). The frequencies of transgressive overyielding were 25%, 25% and 66% for the three (150 mg, 300 mg and 450 mg) biomass levels, respectively. The overall cumulative effect size was significantly positive [mean 0.12 (±95% CI 0.22-0.03)] only at the highest biomass level. Note that at the highest biomass treatment, the frequency of occurrence of transgressive overyielding was higher for the 3-species assemblages (100%) than for the 2-species assemblages (55.5%), suggesting that biomass-mediated complementarity interactions on multiple ecosystem processes are more probable as the number of species increases.

**Figure 3:**
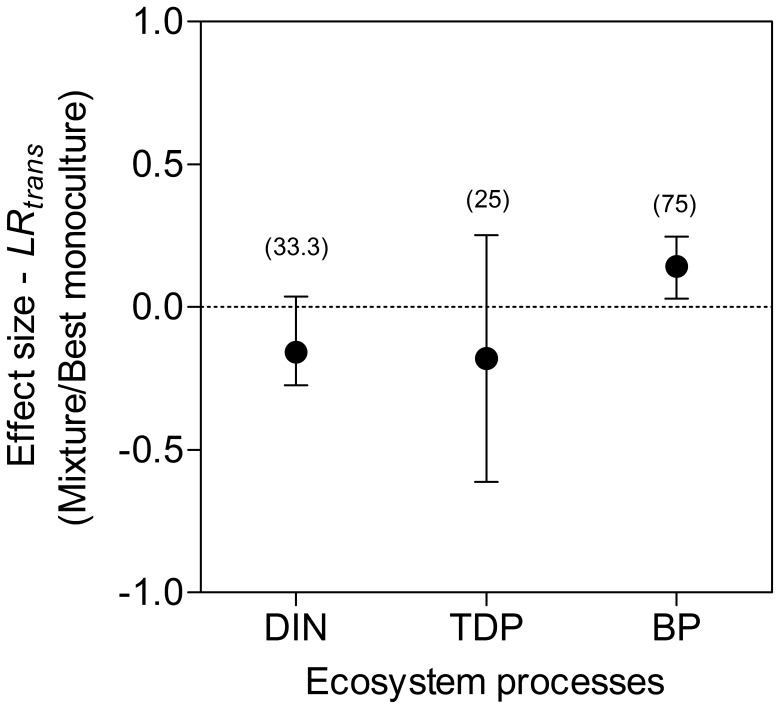
Transgressive overyielding for the ecosystem processes analyzed. Diversity effect sizes (ln response ratios) and their ±95% bootstrapped CI were estimated from the weighted integration, throughout 9999 iterations, of the effect sizes calculated from the proportional response of 2- and 3-species mixtures to their respective best constituent monoculture for each biomass level. Significant overall transgressive overyielding occurs if the value of *LR_trans_* and its confidence interval are greater than zero (dashed line). Abbreviations for ecosystem processes are as in figure 2.

The patterns of frequency and magnitude of transgressive overyielding also varied consistently among ecosystem processes (Fig. 3). The occurrence of complementarity effects was consistent only for BP [mean *LR_trans_* 0.14 (±95% CI 0.24-0.3)], where 75% of the *LR_trans_* values were positive. The analysis of the DIN and TDP fluxes showed that most of the species mixtures (66.7% and 75%, respectively) were not higher than their respective best constituent monoculture and that the cumulative effect sizes were both negative.

**Figure 4:**
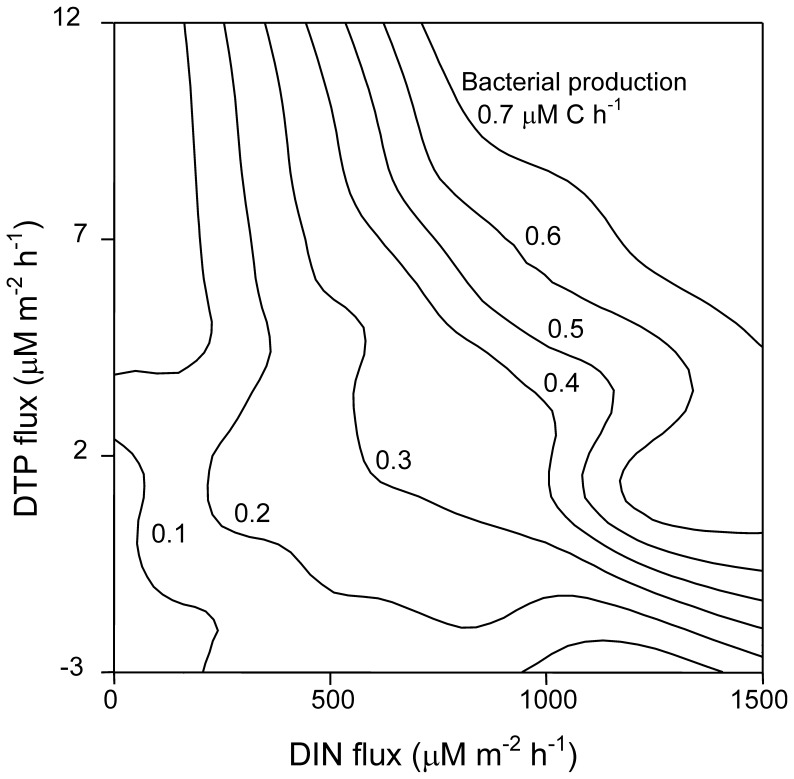
Isopleths showing changes in bacterial production (BP) as a function of dissolved inorganic nitrogen (DIN) and total dissolved phosphorus (TDP) fluxes in microcosms inhabited by benthic bioturbators.

The BP was strongly correlated with the fluxes of DIN and TDP (Table 2; Fig. 4). The best model, selected according to the AIC differences (Δ AIC*_c_*), included both nutrient fluxes and explained approximately 40% of the variation in BP in the experimental chambers. The Akaike weight for this model, relative to the weights for the other two models, is very large. This outcome produces large differences in evidence ratios for the best model against the two other models (w_1_/w_i_ = 249 and >996). In addition, according to Burnham and Anderson [78], models with Δ AIC*_c_* >10 may be discarded due to the low level of empirical support.

**Table 2:** Output of best-model selection procedure for bacterioplankton production based on Akaike selection criterion values (Δ AIC*_c_*).

Model_c_	_1_ *R^2^*	AIC*_c_*	Δ AIC*_c_*	*w*_i_	w_1_/w_i_
DIN, TDP	0.392	−90.473	0	0.996	1
DIN	0.289	−79.65	10.822	0.004	249
TDP	0.174	−67.042	23.431	<0.001	>996

(DIN) Dissolved inorganic nitrogen; (TDP) Total dissolved phosphorus.

Model plausibility decreases with increasing Δ AIC*_c_*. (w_i_) Akaike weight.

## Discussion

Knowledge about BEF has been built primarily upon the statements of resource competition theory [80] and based on single or very few response variables that are very often directly dependent on trophic or consumptive mechanisms (i.e., nutrient or food consumption) [7]. Although resource partitioning has obvious implications for ecosystem processes such as primary production, which depends directly on differences in the way species acquire and convert available resources (e.g., nutrients, water) into biomass, we still have little information on whether and how biodiversity is important for determining complementarity effects on ecosystem processes that are the product of complex non-trophic biogeochemical interactions among species and their environment [14]. This potential role for biodiversity is important because non-trophic ecosystem processes, such as nutrient regeneration by benthic ecosystem engineers, may have independent and/or interactive effects that transcend habitat boundaries [51] and may also not be predicted by classical density-dependent models based on resource competition theory [81], [82]. Our results give evidence that the biodiversity effects of benthic bioturbators on sediment nutrient regeneration can transcend habitat boundaries and affect microbial secondary production in the pelagic habitat. These findings indicate that benthic biodiversity can mediate the bottom-up control of energy flow through pelagic food webs. Furthermore, consistent with our objectives, our work elucidates two seemingly general mechanisms mediating the effects of BEF. First, although the interactive effects between biomass and species richness were not significant for all ecosystem processes, a biomass increase strengthened the complementarity effects of biodiversity if multiple processes were considered together (Fig. 2). Second, according to our predictions, the complementarity effects were stronger on more complex and integrative ecosystem processes and were mediated by multivariate nutrient complementarity. Our data suggest that if individual species have dominant effects on lower-order and relatively “simpler” and subsidiary ecosystem processes (e.g., DIN and TDP fluxes in this study), that interact to determine the magnitude of relatively “more complex and integrative” ecosystem processes (e.g., BP in this study), non-additive effects of biodiversity can be more frequent or stronger on the more integrative ecosystem processes through multivariate nutrient complementarity. These results provide more than an obvious example of how measuring additional response variables can lead to different conclusions about the importance of biodiversity to ecosystem functioning. They highlight the possibility of setting ecosystem processes in a conceptual hierarchical framework according to the number of pathways through which ecosystem processes can be directly and indirectly affected by species actions and their interactions. From this point of view, more complex and integrative ecosystem processes may have an *umbrella* property: it may be more probable that they are affected by the multivariate complementarity effects of species interactions because they depend on the direct and indirect effects of biodiversity on multiple and interacting subsidiary “simpler” ecosystem processes.

### Importance of Community Biomass for the Effects of Bioturbator Diversity on Benthic-pelagic Processes

Given that community diversity and biomass commonly covary in nature, quantifying the effects of BEF across levels of abundance has been recognized to be of paramount importance to the integral recognition of the value and management of biodiversity [17]. At a one-dimensional level, only DTP flux was affected by the biomass × species richness interaction. Interestingly, however, significant complementarity effects occurred for the highest biomass level if the simultaneous effects of biodiversity on multiple processes were considered. This significant overall complementarity effect at the highest biomass level cannot be solely attributed to biodiversity effects on DTP flux because 66% of the diversity effect sizes (8 out of 12) calculated for the highest biomass level indicated the occurrence of transgressive overyielding (Fig. 2). In accordance with recent studies that have shown that structural attributes of ecological communities, such as functional trait diversity and species composition and evenness, determine ecosystem multifunctionality [31], [83], our data show for the first time that community biomass mediates the effects of biodiversity on multiple ecosystem processes. This finding has important functional and conservation repercussions because it indicates that multiple functions of ecosystems may be jeopardized even before species become extinct.

Density has been suggested as an important mechanism affecting the strength of intra- and interspecific interactions in nature because it can mediate patterns of resource use and facilitative interactions [84]. In a broader sense, two classes of non-mutually exclusive mechanisms could explain the biomass-mediated effects of biodiversity observed in our results: a relaxation of intraspecific interference and a differentiation of the use of sedimentary space. Both mechanisms could interact if species show clear differences in the bioturbating domain where they forage. For example, by including species that bioturbate at different depths of the sediment biotope space, more of the sedimentary habitat can be bioturbated and less intraspecific interference would occur on a single portion of the sediment than would result from interference with bioturbation behavior due to biomass increase. Obviously, by increasing biomass we also inevitably increase the probability of interspecific interference, but the outcome of this counteracting effect for the rates of measured processes will depend on the relative importance of intra- and interspecific interactions to these processes. We hypothesized that the increasing biomass of an assemblage of species that diverge substantially in their use of sediment vertical space could generate, at a given point, an “intermediate” type of niche overlap that can be beneficial for enhancing the rates of non-trophic ecosystem processes, such as benthic-pelagic nutrient fluxes. The hypothesis above finds considerable support in the literature. Caliman *et al.* [44], using a community of 3 species of benthic invertebrates that functionally resembles that used for this experiment, demonstrated that the bioturbation-mediated regeneration of nutrients was positively affected by species richness only if the sediment was sufficiently deep to accommodate the complementary use of the entire sedimentary space by species. This result indicates that interspecific interference can explain some reductions in the rates of bioturbation-mediated nutrient fluxes. However, several studies have shown that a given degree of physical proximity among biogenic structures, which can be mediated by invertebrate density [41], [51], is important to generate transient spatial and temporal discontinuities in sediment geochemistry, with further benefits to the microbial mineralization processes within the sediments and, consequently, to the rates of benthic-pelagic nutrient fluxes [85], [86]. The dynamic interdependencies among such non-trophic density-mediated microbial-sediment-macrofauna biogeochemical transformations make them almost virtually impossible to test experimentally [20], but our results suggest that the density-mediated effects of bioturbator biodiversity on non-trophic benthic-pelagic processes depend strongly on the partitioning of the sedimentary habitat among bioturbator species.

### Importance of the Characteristics of Ecosystem Processes to the Occurrence of Multivariate Complementarity

BEF studies have generally neglected the importance of considering interactions among ecosystem processes as possible pathways of the mechanisms driving non-additive effects of biodiversity. Although this neglect is logically affected by the historical overemphasis on testing the effects of biodiversity on single processes, as highlighted before, this source of non-additive effects is frequently observed, even in studies that have measured multiple processes simultaneously [87]–[89]. Even the recent focus on biodiversity effects on ecosystem multifunctionality has neglected the potential importance of considering interactions among ecosystem processes [23]–[25], [31], [76]. In our opinion, this neglect reflects a lack of theoretical development, and this absence of applicable theory prevents BEF studies from considering ecosystem processes as a source of variation rather than simply as a product of variation. The lack of such a conceptual framework can greatly impede our understanding of the importance of biodiversity to ecosystem functioning over larger spatial and temporal scales because the functioning of whole ecosystems is a complex amalgam among multiple processes that are dynamically determined by biotic and abiotic mechanisms that interact across multiple spatial and temporal scales [23], [90].

BP, considered by us as a more complex and integrative ecosystem process than DIN and TDP fluxes, was consistently more subject to complementarity effects, both in terms of the frequency of occurrence and in terms of the overall strength of positive *LR_trans_* (Fig. 3). In addition, species richness was more important than species composition in explaining variation in BP, both in ANOVA and regression models (Table 1, Fig. 1). Our results strongly suggest that the consistent complementarity effect of bioturbator diversity on BP was a product of multivariate nutrient complementarity. This mechanism was apparently mediated by selection effects caused by the dominance effects of different bioturbator species on DIN and TDP fluxes, which were shown to co-limit BP in our experiment (Table 2, Fig. 4). The preponderance of selection effects associated with the species *Chironomus* sp. and *H. similis* on the fluxes of DIN and TDP, respectively, can be indicated by the importance of species composition to both nutrient fluxes and the absence of significant *LR_trans_* for them (Table 1, Fig. 3). Taken together, these results indicate that multivariate complementarity could emerge if different species dominate subsidiary ecosystem processes that are important for determining the rates of more complex integrative ecosystem processes. To the best of our knowledge, our results bear certain similarities with the findings of two previous studies, but important and complementary differences are present as well. Duffy et al., [30] suggested the term *multivariate dominance effect* to describe the phenomenon that even if single species are most important for individual ecosystem processes (i.e., sampling effects), only species mixtures maximize multiple ecosystem responses simultaneously. However, this approach does not consider the multivariate dominance effect as a mechanism mediating hierarchical interactions among ecosystem processes, as indicated by our results. Bracken and Stachowicz [29] showed that because macroalgae appear to differ in the efficiency with which they use different forms of inorganic nitrogen, total nitrogen use is higher in diverse assemblages via multivariate nutrient complementarity. This mechanistic role of multivariate nutrient complementarity is similar to that suggested by our work, but authors disregarded the importance to consider the complexity of the response variable as an interacting factor affecting the effects of biodiversity. Therefore, we believe that our work represents a step forward in the development of BEF research because we consider the complexity of ecosystem processes as a characteristic mediating both the probability of occurrence and the strength of multivariate complementarity.

We recognize the limitations associated with the consideration of the results of a single study in support of the validity of a broad hypothesis. Our hypothesis that complex integrative ecosystem processes better encapsulate multiple mechanisms responsible for generating complementarity effects appears to be reasonable in terms of our results. However, what is the relevance of this hypothesis to the broad context of BEF research? Undoubtedly, better tests of this hypothesis could be conducted with a meta-analysis that would examine multiple studies to determine how the strength of complementarity effects varies according to ecosystem processes differing in complexity. However, evidence from a large-scale BEF study offers substantial general support to our results. Spehn *et al.* [91] analyzed the results of a cross-European experiment that tested the effects of grassland vegetation diversity in terms of several ecosystem functions. These functions ranged from the rates of consumption of specific resources (i.e., subsidiary processes such as soil nitrogen acquisition and light use), to the rates of conversion of resources into biomass (i.e., integrative processes such as aboveground primary productivity). The authors of the study observed that the effect of plant species number was stronger on primary productivity than on ecosystem processes representing resource consumption. These findings offer considerable support to our hypothesis and indicate that BEF research may greatly benefit from a theoretical framework that considers the hierarchical structure in which ecosystem processes are embedded. For example, if integrative ecosystem processes encapsulate the effects of biodiversity on various subsidiary processes, they can be used as proxies of ecosystem multifunctionality. In fact, most of the data that have been used in recent BEF studies designed to demonstrate the effects of biodiversity on ecosystem multifunctionality originate from the experiments that first demonstrated the effects of plant diversity on primary productivity [23], [25], [76] and therefore elicit a possible link between primary production and ecosystem multifunctionality. The possibility of this link is reinforced by the fact that primary production has been considered as a master variable that integrates multiple environmental factors [92] and could therefore explain why primary production has been so responsive to experimental manipulations of species diversity [93]. Finally, another important ramification is that if functional redundancy among species tends to decrease with the number of functions considered [27], [94], then integrative ecosystem processes should be more strongly affected by biodiversity because it may better integrate the contribution of the functional traits of multiple species to ecosystem function across space and time. It is particularly important to predict effects of biodiversity in situations where communities are composed of species that show considerable functional plasticity, such as benthic invertebrate bioturbators [95]–[97].

### Conclusions

In summary, our work showed that non-trophic biogeochemical interactions mediated by benthic bioturbator species can transcend habitat boundaries and ultimately generate higher rates of pelagic microbial production. This result is important because BEF studies have increasingly attempted to demonstrate the importance of biodiversity in a food web perspective [98] and over broad spatial scales [99]. Such findings can be particularly important to the functioning of shallow lakes, where habitat coupling is stronger and benthic-derived subsidies assume greater importance for the function of the entire ecosystem [100]. An even more important aspect of our study is that our results show the significance of considering density-mediated mechanisms and the structural characteristics of the response variables as important factors in explaining the mechanisms that determine the occurrence and strength of complementarity effects of biodiversity. Most BEF studies have emphasized only the worst scenario, which considers the negative effects of species extirpation on ecosystem functioning. However, a decrease in population always precedes the extinction of a species [101]. We show that density-dependent mechanisms can determine the ability of species to interact complementarily so that reductions in species/community biomass can represent a functional loss of biodiversity even before species extinction. Finally, we also show the importance of considering a hierarchical framework that determines potential interactions among multiple ecosystem processes varying in complexity to better understand and predict the complementarity effects of biodiversity. Ecologists have focused on understanding how biodiversity affects ecosystem processes, but we have neglected to consider the innate ability of ecosystem processes to capture biodiversity effects. It is undoubtedly a matter of future research, because the effects of biodiversity on complex and integrative ecosystem processes that are the product of multiple indirect interactions are not necessarily encapsulated by the assumptions of trait-function relationship that underlies current BEF research. We believe that considering hierarchical attributes of ecosystem processes in a functional perspective will greatly improve our ability to understand the mechanisms driving the effects of biodiversity on ecosystem functioning.

## Supporting Information

Figure S1 **Model picture (not a representation of the experimental microcosms used for this study) highlighting the functional differences among the three benthic invertebrate species used for this experiment.** The organisms were placed in a thin aquaria filled with white sand (not the azoic sediment used in the experiment) to improve their visibility. The species show remarkable complementarity in their spatial distribution and foraging behavior within the sediment.
(TIF)
