# Supplementary figures and images for: Community Biomass and Bottom up Multivariate Nutrient Complementarity Mediate the Effects of Bioturbator Diversity on Pelagic Production
Source: PLoS One. 2012 Sep 12;7(9):e44925. doi: 10.1371/journal.pone.0044925 (PMC3440345; doi:10.1371/journal.pone.0044925)

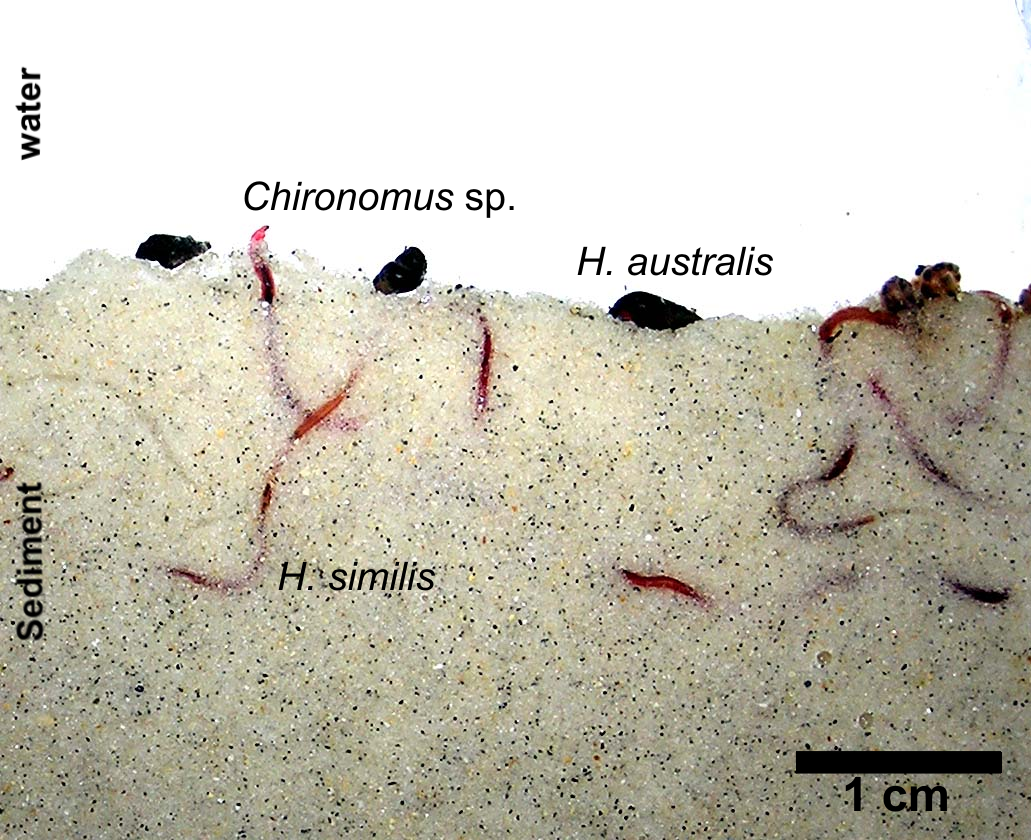

Supplement: Figure S1 — Model picture (not a representation of the experimental microcosms used for this study) highlighting the functional differences among the three benthic invertebrate species used for this experiment. The organisms were placed in a thin aquaria filled with white sand (not the azoic sediment used in the experiment) to improve their visibility. The species show remarkable complementarity in their spatial distribution and foraging behavior within the sediment. (TIF) [file pone.0044925.s001.tif]
